# Identification of the main flavonoids of *Abelmoschus manihot* (L.) medik and their metabolites in the treatment of diabetic nephropathy

**DOI:** 10.3389/fphar.2023.1290868

**Published:** 2024-01-08

**Authors:** Zhipeng Diao, Hongmei Yu, Yapeng Wu, Yuanbo Sun, Haitao Tang, Mei Wang, Nan Li, Haitao Ge, Jianguo Sun, Harvest F. Gu

**Affiliations:** ^1^ Jiangsu Provincial Key Laboratory of Drug Metabolism and Pharmacokinetics, Research Unit of PK-PD Based Bioactive Components and Pharmacodynamic Target Discovery of Natural Medicine of Chinese Academy of Medical Sciences, China Pharmaceutical University, Nanjing, China; ^2^ Laboratory of Molecular Medicine, School of Basic Medicine and Clinical Pharmacy, China Pharmaceutical University, Nanjing, China; ^3^ Suzhong Pharmaceutical Research Institute, Nanjing, China; ^4^ Department of Endocrinology, Jiangsu Province Hospital of Chinese Medicine, The Affiliated Hospital of Nanjing University of Chinese Medicine, Nanjing, China

**Keywords:** *Abelmoschus manihot* (L.) medik, diabetic nephropathy, metabolism, pharmacological mechanism, flavonoids, HPLC-Q-TOF-MS/MS

## Abstract

**Introduction:** Huangkui capsule (HKC) is made from the ethanol extract of *Abelmoschus manihot* (L.) Medik [Malvaceae; abelmoschi corolla] and received approval from the China Food and Drug Administration (Z19990040) in 1999. Currently, HKC is used for treatment of the patients with diabetic nephropathy (DN) in China. The bioactive chemical constituents in HKC are total flavonoids of *A. manihot* (L.) Medik (TFA). The present study aims to identify the primary flavonoid metabolites in HKC and TFA and their metabolism fates in db/db mice, the animal model for the study of type 2 diabetes and DN.

**Methods:** HKC (0.84 g/kg/d) and TFA (0.076 g/kg/d) or vehicle were respectively administered daily via oral gavage in db/db mice for 4 weeks. The metabolism fate of the main metabolites of HKC in serum, liver, kidney, heart, jejunum, colon, jejunal contents, colonic contents, and urine of db/db mice were analyzed with a comprehensive metabolite identification strategy.

**Results and Discussion:** In db/db mice administered with HKC and TFA, 7 flavonoid prototypes and 38 metabolites were identified. The related metabolic pathways at Phases I and II reactions included dehydroxylation, deglycosylation, hydrogenation, methylation, glucuronidation, sulphation, and corresponding recombined reactions. Quercetin, isorhamnetin, quercetin sulphate, quercetin monoglucuronide, and isorhamnetin monoglucuronide presented a high exposure in the serum and kidney of db/db mice. Thereby, the present study provides a pharmacodynamic substance basis for better understanding the mechanism of *A. manihot* (L.) Medik for medication of DN.

## 1 Introduction

Diabetic nephropathy (DN) is a complex and long-term kidney disease that develops in individuals with diabetes and is mainly characterized by progressive loss of glomerular function, renal fibrosis, and proteinuria (Thomas et al., 2015; Doshi and Friedman, 2017a; Koye et al., 2018). Consequently, DN has become the leading cause of end-stage renal disease (ESRD), and the patients with ESRD need dialysis or kidney transplantation to stay alive (Doshi and Friedman, 2017b). Due to the numerous influencing factors and complex pathological mechanisms of DN (Gu, 2019; Selby and Taal, 2020), there is a lack of specific drugs for the treatment of DN in clinics. In recent years, natural products have been reported to play an important role in inhibiting the development of DN (Zhong et al., 2015). A recent multicenter, double-blind, and parallel-controlled clinical trial has demonstrated that the application of Huangkui capsule (HKC) and its combination with Irbesartan significantly reduce albuminuria in patients with DN (Zhao et al., 2022).

HKC is a traditional Chinese patent medicine derived from *Abelmoschus manihot* (L.) Medik [Malvaceae; abelmoschi corolla] through a process of ethanol extraction. HKC has received approval from the China Food and Drug Administration (Z19990040) in 1999. The main constituents in HKC are flavonoids, mainly including rutin, hyperoside, hibifolin, isoquercetin, myricetin, quercetin and quercetin-3-O-robinobioside (Guo et al., 2015; Li et al., 2021). Previous pathophysiological studies have reported that total flavonoids of *A. manihot* (L.) Medik (TFA) can inhibit microinflammation, prevent kidney damage and podocyte apoptosis, and reduce albuminuria in the early stage of DN (Zhou et al., 2012; Tu et al., 2020; Liu et al., 2021a). Several pharmacological studies have focused on hypericin and suggested its presence in HKC may play a key role in the inhibition of renal inflammation by regulating macrophage polarization and help alleviate the symptoms of early proteinuria by improving glomerular basement membrane damage and podocyte damage in DN (An et al., 2017; Liu et al., 2021b; Zhou et al., 2021). However, it is still unclear what are the flavonoids and their related metabolites responsible for the pharmacological effects in DN.

The metabolism of traditional Chinese medicine is closely related to its pharmacological activity. Our research group has recently analyzed not only the microbiota from three parts of the intestines (duodenum, ileum, and colon) but also the metabolites in blood circulation in NOD and db/db mice (an animal model for type 1 and type 2 diabetes, respectively) with and without HKC treatment. The data have implicated that the gut microbiome and circulating metabolomics interact in these animal models while HKC can modulate gut microbiota and subsequently ameliorate the metabolite levels in DN (Wu et al., 2022; Shi et al., 2023). Furthermore, we have demonstrated that *A. manihot* (L.) Medik has pharmacological efficacy in the regression of the development of DN via the regulation of solute carriers in proximal and distal convoluted tubules of kidneys (Yu et al., 2023). The changed microbial flora, altered metabolites, and dysfunction of genes in kidneys may significantly affect the absorption, distribution, metabolism, and excretion of drugs. Therefore, it is necessary to systematically study the exposure components and metabolic characteristics of HKC and TFA *in vivo*, and to screen the pharmacodynamic substance basis of TFA in treatment of DN.

In the present study, we first applied either HKC or TFA for the treatment of db/db mice. We then systematically identified the main constituents and their metabolites in serum, intestinal contents, urine, kidney, heart, liver, jejunum, and colon tissues of the db/db mice after oral administration of HKC or TFA. Finally, all metabolites were quantitatively analyzed based on their peak areas obtained at LC-Q-TOF/MS mass spectrometry, helping to predict highly exposed components or metabolites in target organs. Thereby, the findings from the current study may provide a basis not only for identification of true bioactive components but also for understanding pharmacological mechanisms underlying the use of *A. manihot* (L.) Medik as a medication for DN.

## 2 Materials and methods

### 2.1 Drug and reagents

HKC and TFA were supplied by Suzhong Pharmaceutical Group Co., Ltd. (Batch21122403). As previously reported (Yang et al., 2018), TFA had been further optimized the extraction and purification process based upon HKC, and had a higher content of flavonoids. One Gram of TFA was made from 46.01 g *A. manihot* (L.) Medik flower. *Abelmoschus manihot* (L.) Medik flower was leakaged with 70% ethanol and collected the leachate. The ethanol was recycled and concentrated to a moderate amount. The leachate was extracted with ethyl acetate, and the ethyl acetate extract was collected. The ethyl acetate was recovered under pressure, the D101 macroporous resin column was diluted with water and then eluted with 4BV water, 4BV 5%, and 4BV 60% ethanol. The 60% ethanol eluent was collected and the ethanol was recovered, vacuumed dry and pulverized. TFA was a brown powder, which contains 2.4 mg of rutin, 189.6 mg of hyperoside, 188.7 mg of hibifolin, 142.9 mg of isoquercetin, 33.2 mg of myricetin, 29.4 mg of quercetin, 133.0 mg of quercetin-3-O-robinobioside and 2.7% water per Gram calculated as the dry product the powder of per Gram. In addition, quercetin, myricetin, hyperoside, quercetin-3′-glucoside were supplied by Suzhong Pharmaceutical Group Co., Ltd. Hibifolin, rutin, isoquercitrin, isorhamnetin, and DOPAC were purchased respectively from Selleck and MCE, Shanghai, China.

The HPLC-grade methanol and acetonitrile used in this study were provided by Merck (Darmstadt, Germany), while the HPLC-grade formic acid was obtained from Sigma-Aldrich Chemicals (St. Louis, MO). For the preparation of HPLC-grade ultrapure water, the Milli-Q system was used. All other materials and reagents utilized in this study were of analytical purity available commercially.

### 2.2 Chemical analysis of TFA

A total of 50 mg of HKC contents and 20 mg of TFA were weighed into a 1.5 mL EP tube, respectively, and extracted with 1 mL of 75% methanol for 30 min, the mixture was centrifuged at 18,000 rpm for 5 min subsequently. 10 μL of the supernatant was diluted with 990 μL of methanol and centrifuged at 18,000 rpm for 5 min. After centrifugation, 100 μL of the supernatant (0.5 mg/mL and 0.2 mg/mL) was extracted and transferred into autosampler vials to be used for HPLC-Q-TOF/MS analysis. The quantitative analyses of seven flavonoids in the flowers of *A. manihot* (L.) Medik. were done according to the previous study (Lu et al., 2013). Preparation of standard stock solution: correctly weighed to obtain quercetin, myricetin, hyperoside, quercetin-3′-glucoside, hibifolin, rutin, and isoquercitrin standard. Then, methanol was added, and the mixture was sonicated to dissolve it. Added more diluent until the desired level was reached, then mixed thoroughly.

The separation was performed on an ACQUITY UPLC BEH C18 column (2.1 mm × 100 mm, 1.7 μm) with 0.05% formic acid-water (A)-acetonitrile (B) as the mobile phases at a flow rate of 0.40 mL/min, and the elution was performed by a gradient elution (0–2 min, 88%A→85%A; 2–5 min, 85%A→84%A; 5–6 min, 84%A→83%A; 6–9 min, 83%A→70%A; 9–11 min, 70%A→20%A; 11–12 min, 20%A→5%A; 12–13 min, 5%A→88%A) at a wavelength of 360 nm, and a column temperature of 35°C. The injection volume was 10 μL.

### 2.3 Animal studies

#### 2.3.1 Animal experimental design and administration

Db/db (BKS.Cg-Dock7m +/+ Leprdb/J) mice are commonly used as animal models for the study of T2D and DN (Sharma et al., 2003). In our study, male db/db and C57BL/6J mice at 8 weeks old were purchased from Animal Experimental Center, Nanjing University (Nanjing, China). The mice used in this study were housed in a specific pathogen-free (SPF) barrier environment located at the Animal Experimental Center of China Pharmaceutical University. The animal room was maintained at a temperature of 24°C ± 2 and a humidity level of 50% ± 10%, with a 12-h light/12-h dark cycle. All experiments in animals were conducted in compliance with the principles of the declaration of Helsinki and approved by the ethics committee of CPU (Approval Code: 2019-08-0003 and Approval Date: 2019-08–26).

After 1-week adaption, the db/db mice were categorized into 3 groups, i.e., DN without treatment (n = 6), HKC treatment (n = 6), and TFA treatment (n = 6). One HKC contains 0.43 g of *A. manihot* (L.) Medik extracts and is produced by Suzhong Pharmaceutical Group Co., Ltd. (Taizhou, China). As we have recently reported (Yu et al., 2023), HKC was dissolved in pure water and freshly prepared as HKC suspension for use. The dosage of the HKC treatment group is 0.84 g/kg/day, converted based on clinical dosage. The administration period was 4 weeks. TFA was prepared from HKC and then dissolved in distilled water and freshly prepared for use. In the TFA group, TFA (0.076 g/kg/d) was administered via oral gavage for 4 weeks. In the control group, distilled water at the same volume as the HKC group was used. In addition, C57BL/6J mice were used as the non-diabetic control group (Control, n = 6). Body weight and blood glucose levels were examined weekly. Urine samples were obtained using metabolic cages. (DXL-XS, Fengshi, Suzhou, China). Microalbuminuria (MAU) and creatinine (Cr) were measured by using ELISA quantitative kits (Elabscience Biotechnology, China). Blood glucose levels ≥16.7 mmol/L and urinary albumin-to-creatinine ratio (UACR) ≥200 ng/μg for two successive days among db/db mice were diagnosed as DN.

#### 2.3.2 Collection of biological samples

After HKC or TFA treatment for 4 weeks, the db/db mice were anesthetized by intraperitoneal injection of 1% pentobarbital sodium. Blood samples from the eyes were collected and placed in the 2 mL collection tube. The serum was then extracted with a centrifuge at 2000 rpm for 20 min. After that these mice were sacrificed to collect tissue samples. The middle lobe of the liver, heart, and right kidney of the mice were selected for sampling and stored in liquid nitrogen after being removed fat with tweezers. The 8 cm length of the jejunum and colon were ligated, and the intestinal contents were not exposed to air by using surgical knotting. All obtained biological samples were preserved at −80°C for experimental use.

### 2.4 Analysis of flavonoid constituents’ metabolites *in vivo*

#### 2.4.1 Processing of biological samples

Serum samples (300 μL per group) from Control, HKC, and TFA groups were pipetted in centrifuge tubes, respectively. The methanol containing 50 ng/mL naringin (Internal standard, IS) at 5 times of volume was then added into the tubes and vortexed for 5 min, subsequently subjected to centrifugation at 18,000 rpm for 5 min. All the supernatants were dried by a vacuum evaporator. 80 μL 50% methanol was used to reconstitute the residues and then centrifuged as described above. Finally, 70 µL of supernatants were used for LC-Q-TOF-MS analysis.

The samples of tissues, including the kidney, heart, liver, jejunum, and colon, as well as the samples of intestinal contents were homogenized with 10 times of volume of physiological saline water (100 mg/mL). These homogenate samples (1.2 mL per group) were then pipetted into centrifuge tubes. Then, methanol containing naringin (IS, 50 ng/mL) at 5 times of volume was added and vortexed for 5 min, and subsequently subjected to centrifugation at 18,000 rpm for 5 min. All the supernatants were dried by a vacuum evaporator. 80 μL 50% methanol was used to reconstitute the residues and then centrifuged as described above. Finally, 70 µL of supernatants were used for LC-Q-TOF-MS analysis.

#### 2.4.2 HPLC-Q-TOF-MS/MS analytical conditions

In the current study, a TOF-MS/MS method was developed to detect flavonoid constituents and their metabolites in db/db mice. A Shimadzu LC-30A system (Kyoto, Japan) was used in this analysis. The mobile phase consisting of mobile phase A (0.1% formic acid-water) and mobile phase B (acetonitrile) was used for separation on an XSelect HSS T3 column (2.1 × 150 mm, 3.5 μm) with a flow rate of 0.3 mL/min. The following gradient elution program was used: 0–2 min, 5% B; 2–7 min, 9%–40% B; 7–12 min, 40%–65% B; 12–15 min, 65% B; 15–17 min, 65%–90% B; 17–20 min, 90% B; 20–22 min, 90%–5% B and 22–25 min, 5% B. The injection volume was 10 μL.

Mass spectrum data were acquired on AB Sciex 5600 Q-TOF mass spectrometer (Framingham, MA, United States) using negative information-dependent acquisition (IDA) modes. The source parameters were optimized and set as follows: MS and MS/MS scan mass range, *m/z* 100–1200 Da; gas 1, 60 Arb; gas 2, 65 Arb; curtain gas, 35 Arb; ion spray voltage, −4500 V; temperature, 550 °C; DP, −100 V; CE, −10 eV for MS scan, and 55 ± 25 eV for MS/MS scan. Real-time calibration was achieved by injecting APCI calibration solutions for every 5 samples. Analyst TF software (AB SCIEX) was used to acquire MS data.

#### 2.4.3 Analytical strategy of metabolites

**FIGURE 1:**
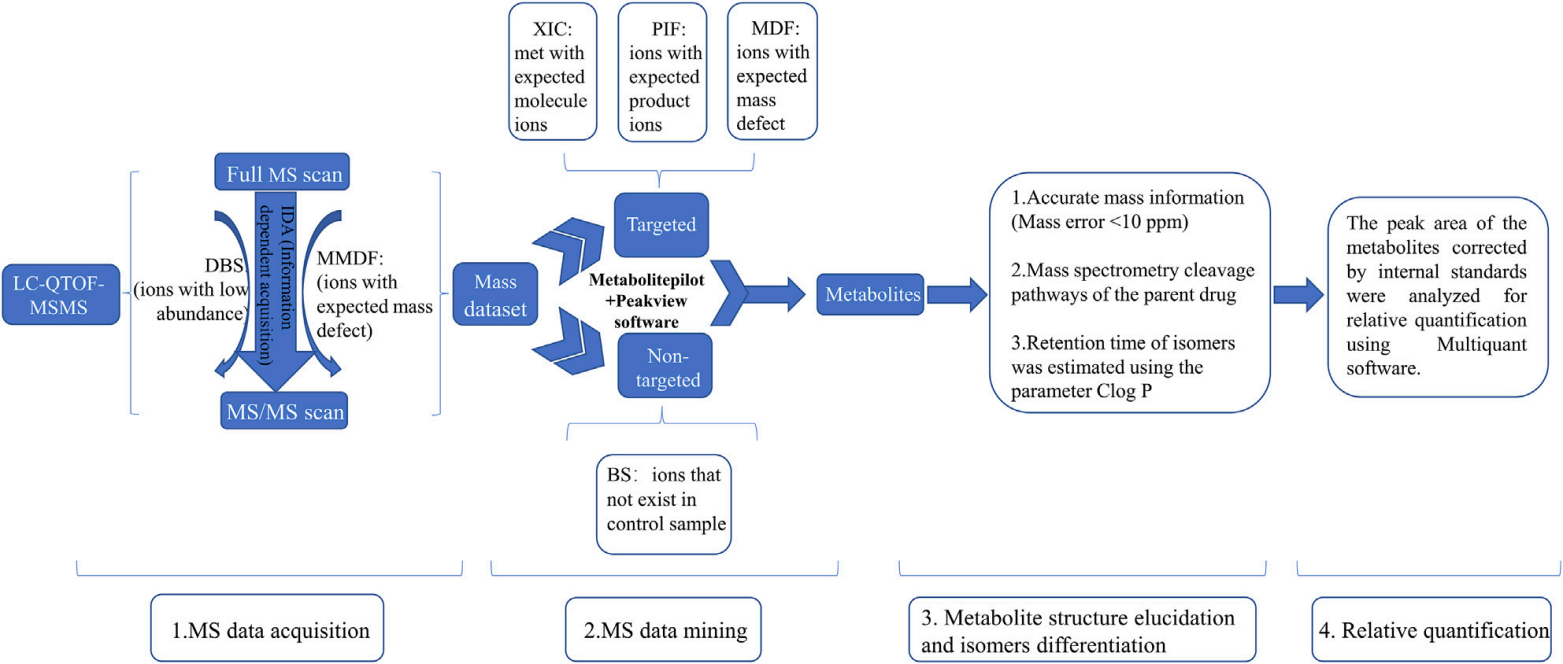
The strategies for identification of metabolites of *Abelmoschus manihot* (L.) Medik DBS: dynamic background substract; MMDF: multi mass defect filtering; XIC: extracted ion chromatogram; PIF: product ion filter; BS: background subtraction.

A previous study has suggested that the trace metabolites in full-scan mass chromatograms may be overwhelmed by interference from the matrix, resulting in their MS^2^ spectrum acquisition not being triggered (Liu et al., 2014). To detect as many metabolites as possible, as shown in Figure 1, we used an IDA acquisition strategy based on multi mass defect filtering (MMDF) and dynamic background substract (DBS), which can trigger IDA scan to detect low-intensity metabolites obscured by background noise. For mass spectrometry data post-processing, we applied various data processing techniques provided by MetabolitePilot™ software (AB Sciex), such as extracted ion chromatogram (XIC), mass defect filtering (MDF), and product ion filtering (PIF), to help rapidly mine possible metabolites from the massive mass spectrometry data. Then, accurate mass information (Mass error <10 ppm) and mass spectrometry cleavage pathways of the parent drug help to further elucidate metabolite structures. Meanwhile, the retention time of isomers was estimated using the parameter Clog *P*. In general, metabolites with larger Clog *P* usually have longer retention time in reversed-phase liquid chromatography systems (Zhang et al., 2017). Finally, the peak areas of the metabolites corrected by internal standards were analyzed for relative quantification using Multiquant software (AB Sciex).

### 2.5 Statistical analysis

Statistical analysis was conducted using GraphPad Prism 8.0 software (La Jolla, United States). To examine the differences among the groups, one-way analysis of variance (ANOVA) followed with Bonferroni *post hoc* analysis was used. The data were presented as mean ± SEM. The *p*-value <0.05 was considered statistically significant.

## 3 Results

### 3.1 Reduction of UACR after treatment of HKC and TFA

**FIGURE 2:**
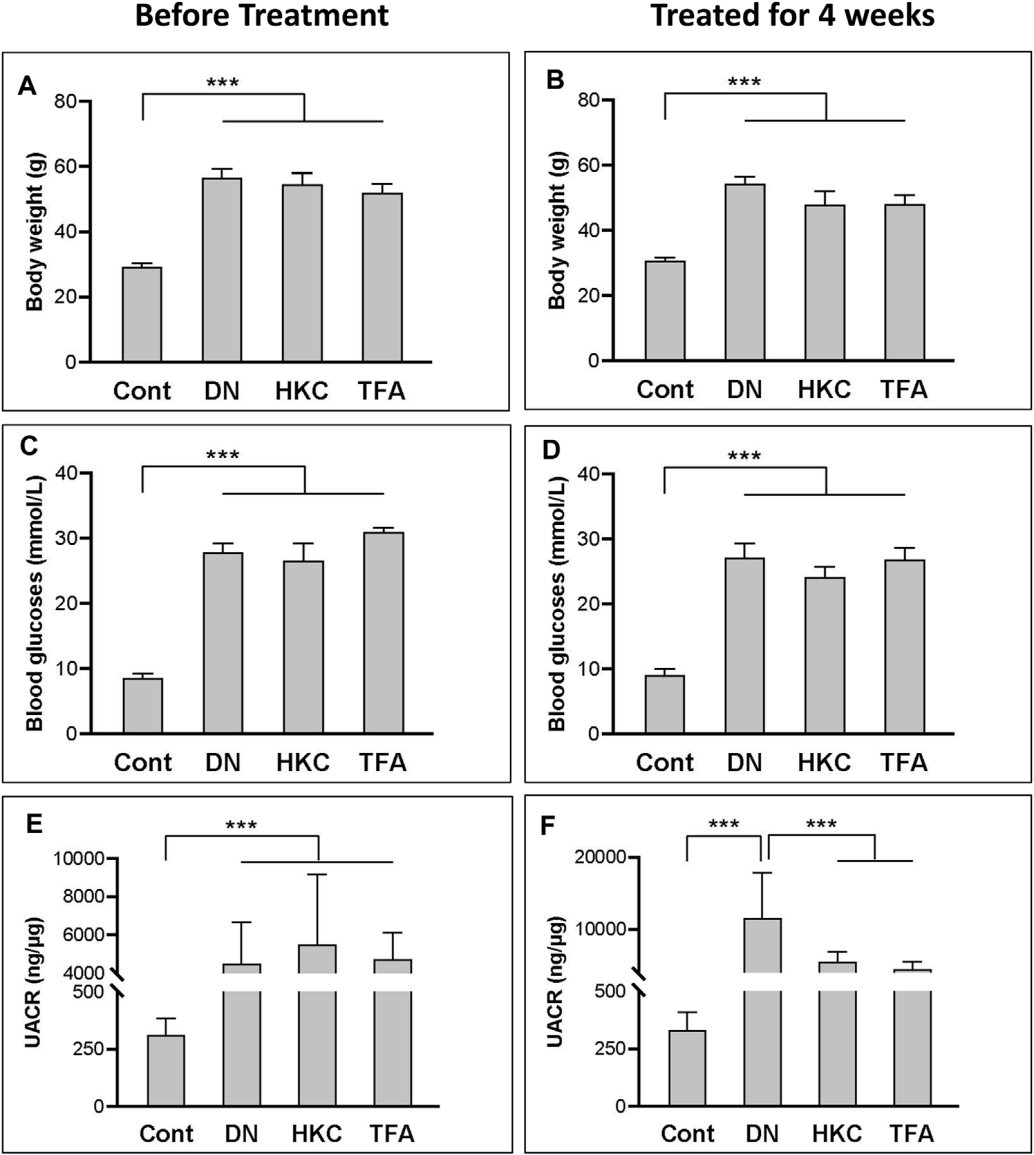
The changes of blood glucose levels, body weight and UACR in db/db mice before and after HKC and TFA treatment Body weight **(A,B)**, blood glucose levels **(C,D)** and UACR **(E,F)** in non-diabetic controls, db/db mice before and after HKC and TFA treatment for 4 weeks. HKC, db/db mice with Huangkui capsule treatment; TFA, db/db mice with the administration of total flavonoids of *Abelmoschus manihot* (L.) Medik*;* UACR, urinary albumin-to-creatinine ratio; ***p < 0.001.

In the whole procedure of experiments, body weight, blood glucose levels, and UACR were examined. Data demonstrated that the db/db mice with DN had significantly increased body weight, blood glucose levels, and UACR than what in the mice of Control. group (Figures 2A, C, E). After treatment with either HKC or TFA, body weight and blood glucose levels of the db/db mice had no significant changes compared to the same mice before treatment (Figures 2B, D). However, UACR in the db/db mice was found to be significantly decreased after HKC and TFA treatment (Figures 2E, F). Besides, HKC and TFA treatments showed similar reductions in proteinuria with no significant differences (Figure 2F).

### 3.2 Identification of flavonoid metabolites in HKC and TFA

**FIGURE 3:**
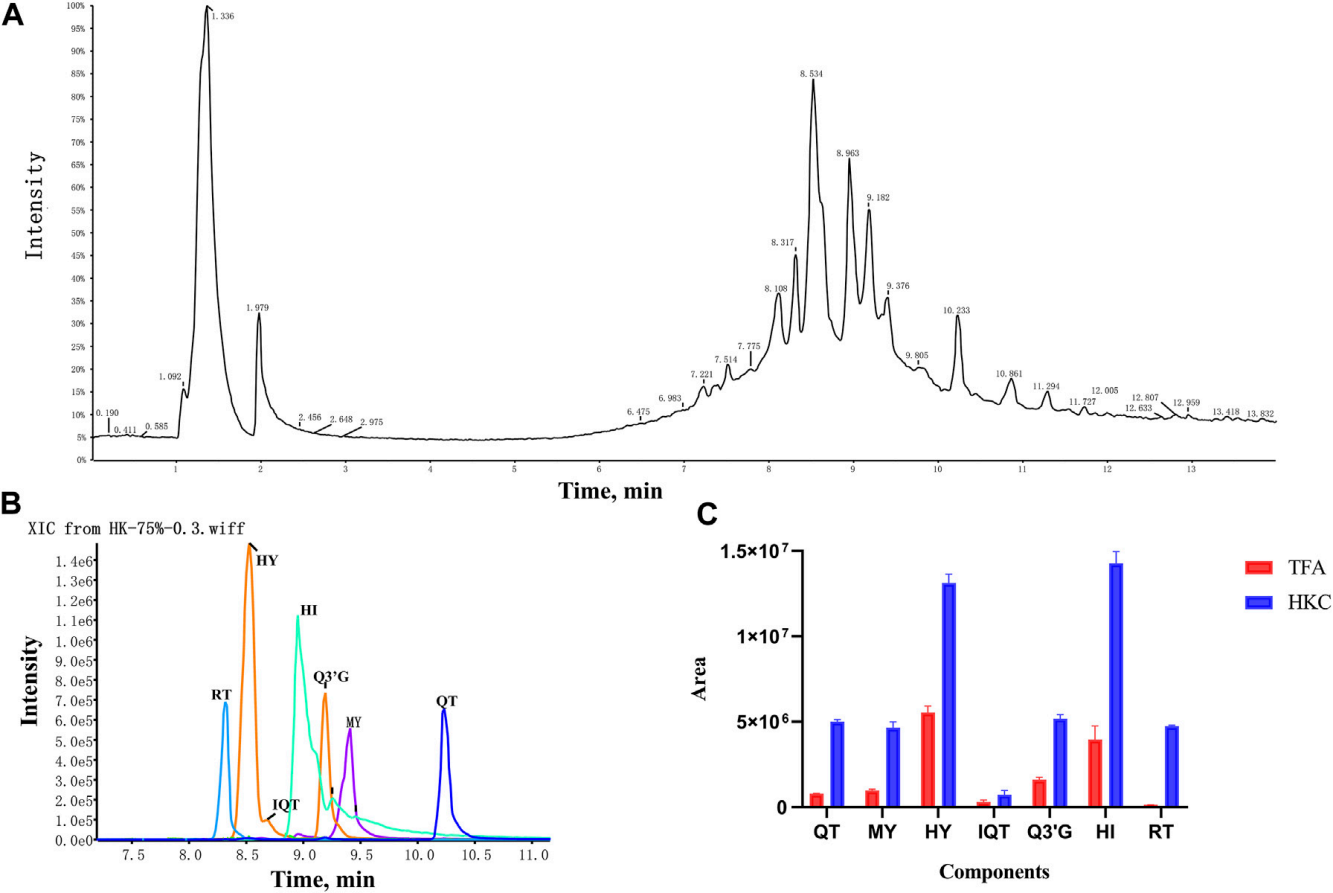
The TIC, XIC and relative content of each flavonoid constituent in HKC and TFA **(A)**, The total ion chromatogram of HKC; **(B)**, The extracted ion chromatogram of quercetin, myricetin, hyperoside, isoquercitrin, quercetin-3′-glucoside, hibifolin, and rutin in HKC. **(C)**, The relative content of each flavonoid constituent in HKC and TFA. HKC, Huangkui capsule treatment; TFA, total flavonoids of *Abelmoschus manihot* (L.) Medik*;* QT, quercetin; MY, myricetin; HY, hyperoside; IQT, isoquercitrin; Q3′G, quercetin-3′-glucoside; HI, hibifolin; RT, rutin.

A total of seven flavonoid metabolites with high content were identified in 75% methanol extract of HKC and TFA, which were verified by the standards and databases. The relative content of each metabolite is shown in Figure 3. The retention time and secondary fragmentation ions of each monomer under the chromatographic conditions selected in this study are shown in Table 1, and the extracted ion chromatograms, secondary mass spectra, and inferred mass spectral cleavage pathways of each monomer are shown in Supplementary Figure S1. The mass spectrometric cleavage patterns of each monomeric component laid the foundation for the structural speculation of other metabolites.

**TABLE 1:** HPLC-Q-TOFMS analysis of the main flavonoid monomer components of HKC and TFA.

No	ID	*t*_*R*_/min	Molecular	Ion mode	Observed *m/z*	Calculated *m/z*	Error	MS/MS (*m/z*)
1	quercetin	10.5	C_15_H_10_O_7_	[M-H]-	301.0347	301.0354	−2.3	301.0361; 273.0405; 178.9990; 151.0042; 121.0291; 107.0133
2	myricetin	9.67	C_15_H_10_O_8_	[M-H]-	317.0295	317.0303	−2.5	317.0293; 271.0243; 199.0425; 178.9963; 167.0022; 151.0019; 139.0049; 111.0094
3	hyperoside	8.54	C_21_H_20_O_12_	[M-H]-	463.0856	463.0882	−5.6	463.0893; 301.0377; 271.0242; 255.0297; 243.0311; 151.0034
4	isoquercitrin	8.68	C_21_H_20_O_12_	[M-H]-	463.0855	463.0882	−5.8	463.0893; 301.0377; 271.0242; 255.0297; 243.0311; 151.0034
5	quercetin-3′-glucoside	9.3	C_21_H_20_O_12_	[M-H]-	463.0862	463.0882	−4.3	463.0994; 301.0394; 271.0289; 178.9999; 151.0034
6	hibifolin	9.2	C_21_H_18_O_14_	[M-H]-	493.0616	493.0624	−1.6	493.0585; 317.0307; 299.0199; 194.9936; 166.9986; 139.0036
7	rutin	8.54	C_27_H_30_O_16_	[M-H]-	609.1464	609.1461	0.5	609.1443; 301.0345; 271.0259; 255.0326; 178.9999; 151.0039

### 3.3 Establishment of MMDF and DBS-based IDA acquisition method for metabolite analysis

In our study, the IDA acquisition method based upon MMDF and DBS was used to mine all possible metabolites. The setting of the template of MMDF was based upon the properties of the parent drug and the general metabolite biotransformation pattern. The seven major flavonoids in TFA had three parent core structures, quercetin, myricetin, and gossypetin. Based on the 3 core structures and the main biotransformation pathways reported in the literature: methylation, glucuronidation, and sulfation, 12 templates were used to screen the metabolites of the seven flavonoids, and each MMDF template was set to ±100 mD, as shown in Table 2. Meanwhile, DBS can also trigger an IDA scan for acquiring MSMS ions, which is helpful to detect low-intensity metabolites obscured by background noise. Finally, an IDA acquisition method based on MMDF and DBS was established and successfully applied to the metabolite investigation of the seven flavonoids in TFA.

**TABLE 2:** MMDF templates used in IDA acquisition.

Metabolite templates	Formula	MW (Da)	Width (Da)	Mass defect (mDa)	
QT	C_15_H_10_O_7_	302.04	100	42.1	Tolerance: ±100 mDa
QT + CH_2_	C_16_H_12_O_7_	316.06	100	57.8	
QT + SO_3_	C_15_H_10_O_10_S	382	100	998.9	
QT + C_6_H_8_O_6_	C_21_H_18_O_13_	478.07	100	74.2	
MY	C_15_H_10_O_8_	318.04	100	37.0	
MY + CH_2_	C_16_H_12_O_8_	332.05	100	52.7	
MY + SO_3_	C_15_H_10_O_11_S	397.99	100	993.8	
MY + C_6_H_8_O_6_	C_21_H_18_O_14_	494.07	100	69.1	
HI	C_21_H_18_O_14_	494.07	100	69.7	
HI + CH_2_	C_22_H_20_O_14_	508.09	100	85.3	
HI + SO_3_	C_21_H_18_O_17_S	574.03	100	26.5	
HI + C_6_H_8_O_6_	C_27_H_26_O_20_	670.10	100	101.7	

QT, quercetin; MY, myricetin; HI, hibifolin

### 3.4 Identification of flavonoid-related metabolites in db/db mice

**FIGURE 4:**
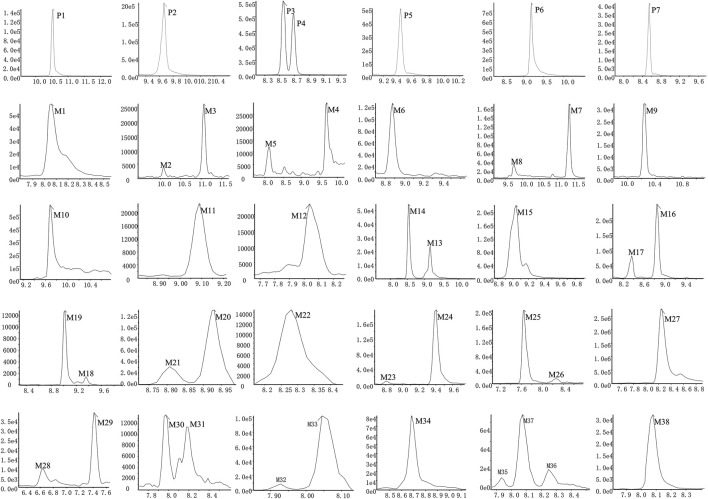
XICs of the flavonoid constituent and its metabolites in db/db mice after oral administration of HKC or TFA HKC, Huangkui capsule treatment; TFA, total flavonoids of *Abelmoschus manihot* (L.) Medik*;* XICs, extracted ion chromatograms.

**FIGURE 5:**
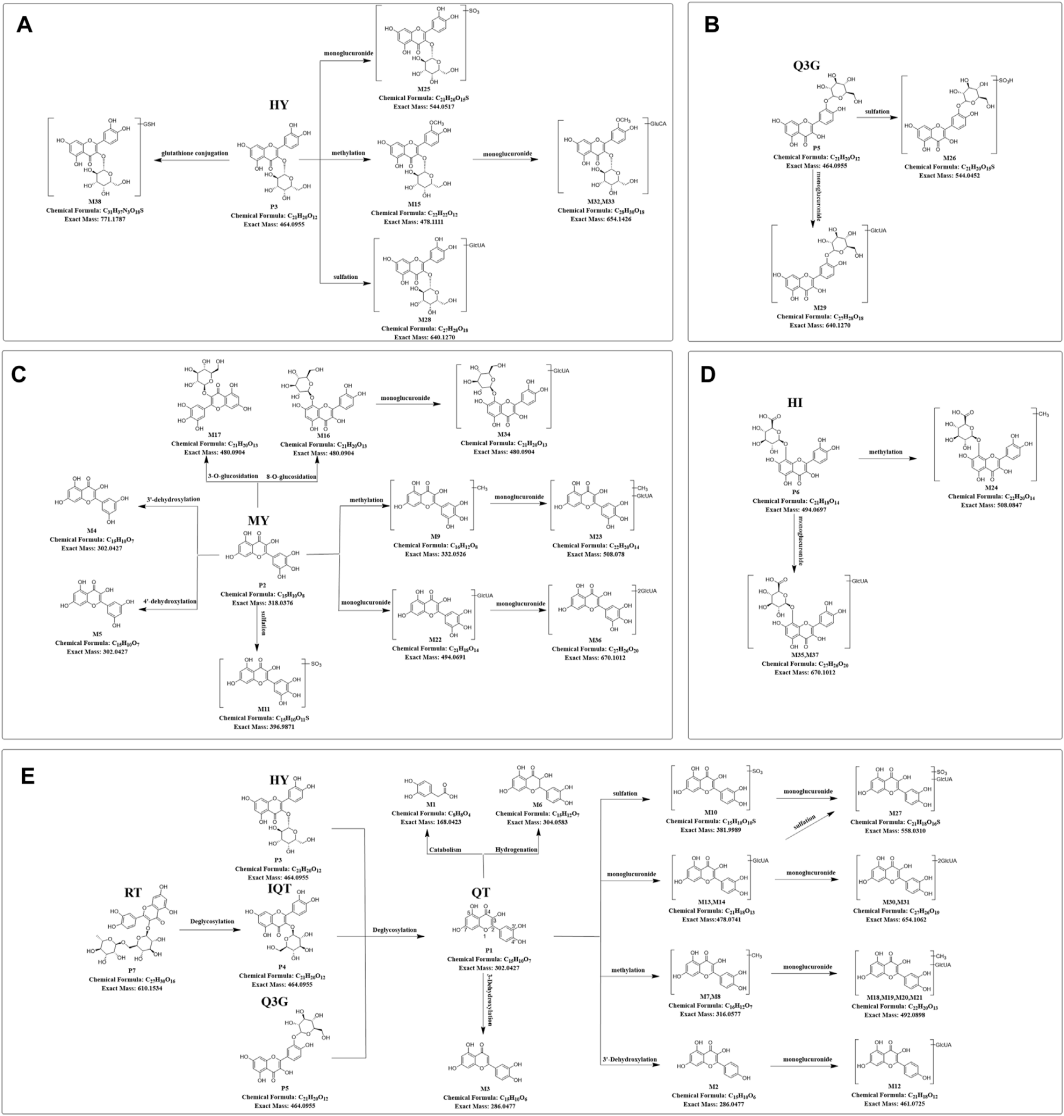
Predicted metabolic pathways of TFA in treatment of db/db mice **(A-E)**, Predicted metabolic pathways of hyperoside, quercetin-3′-glucoside, myricetin, hibifolin, rutin, isoquercitrin, quercetin in db/db mice. TFA, total flavonoids of *Abelmoschus manihot* (L.) Medik*;* QT, quercetin; MY, myricetin; HY, hyperoside; IQT, isoquercitrin; Q3′G, quercetin-3′-glucoside; HI, hibifolin; RT, rutin.

Based upon this strategy, there were 38 metabolites tentatively identified in biological samples including serum, heart, liver, kidney, jejunum, colon, jejunal contents, colonic contents, and urine in db/db mice. The XIC chromatograms of the relevant metabolites and predicted metabolic pathways are represented in Figures 4, 5, respectively. The data suggested that the flavonoids underwent extensive metabolic reactions *in vivo* including dehydroxylation, methylation, sulfation, glucuronidation, glutathione conjugation, deglycosylation, and corresponding recombination reactions. The specific result data were summarized in Table 3.

**TABLE 3:** HPLC-Q-TOF/MS analysis of the metabolites of the flavonoid of *Abelmoschus manihot* (L.) Medik in db/db mice.

NO	*t*_*R*_/min	Molecular	Ion mode	Observed *m/z*	Calculated *m/z*	Error	MS/MS (*m/z)*	Clog P	Metabolite description	Source									Ref
										SE	H	li	k	co	je	U	CO-N	JE-N	
P1	*10.17*	C_15_H_10_O_7_	[M-H]^−^	301.0354	301.0354	0.00	273.0405;178.9988;151.0035;121.0292;107.0144	1.50	quercetin			+	+	+	+	+	+	+	Guo et al. (2011a)
P2	9.32	C_15_H_10_O_8_	[M-H]^−^	317.0314	317.0303	3.47	271.0220;227.0372;178.9986;166.9938;151.0039;137.0247;109.0296	0.84	myricetin					+	+	+	+	+	Guo et al. (2011a)
P3	8.54	C_21_H_20_O_12_	[M-H]^−^	463.0876	463.0882	−1.30	301.0296;271.0263;255.0309;151.0043	−0.34	hyperoside	+	+	+	+	+	+	+	+	+	Guo et al. (2011a)
P4	8.68	C_21_H_20_O_12_	[M-H]^−^	463.0876	463.0882	−1.30	301.0296;271.0263;255.0309;151.0043	−0.34	isoquercitrin							+			Guo et al. (2011a)
P5	9.2	C_21_H_20_O_12_	[M-H]^−^	463.0876	463.0882	−1.30	301.0366;178.9991;151.0043	−0.13	quercetin-3'-glucoside	+		+	+			+	+		Guo et al. (2011a)
P6	8.95	C_21_H_18_O_14_	[M-H]^−^	493.0623	493.0624	−0.20	317.0313;299.0199;271.0235;194.9989;166.9985;139.0043	−1.30	hibifolin		+	+	+	+	+	+	+	+	Guo et al. (2011a)
P7	8.2	C_27_H_30_O_16_	[M-H]^−^	609.1454	609.1461	−1.15	609.1462;301.0369;271.0259;178.9987;151.0033	−1.36	rutin		+	+			+		+	+	Guo et al. (2011a)
M1	8.05	C_8_H_8_O_4_	[M-H]^−^	167.0355	167.0349	3.59	108.0202;91.0195	0.15	DOPAC : 3,4-dihydroxyphenylacetic acid		+		+			+	+	+	Tang et al. (2016)
M2	*10.13*	C_15_H_10_O_6_	[M-H]^−^	285.0405	285.0406	−0.35	217.0482;199.0411;175.0413;151.0076;133.0293	2.10	3'-dehydroxylated quercetin				+	+		+	+	+	Du et al. (2017)
M3	*11.06*	C_15_H_10_O_6_	[M-H]^−^	285.0409	285.0406	1.05	243.0317;229.0498;211.0329;187.0382;167.0493;159.0450;143.0508;108.0199	2.31	3-dehydroxylated quercetin		+	+	+	+	+	+	+	+	Du et al. (2017)
M4^a^	9.71	C_15_H_10_O_7_	[M-H]^−^	301.0356	301.0358	−0.66	178.9977;151.0027;121.0309;107.0129	1.50	3'-dehydroxylated myricetin			+	+		+	+	+		/
M5^a^	*8.24*	C_15_H_10_O_7_	[M-H]^−^	301.0359	301.0354	1.66	178.9977;151.0027;121.0309;107.0129	1.43	4'-dehydroxylated myricetin	+						+		+	/
M6^a^	*8.87*	C_15_H_12_O_7_	[M-H]^−^	303.0515	303.0510	1.65	285.0379;241.0491;217.0519;199.0392;175.0405;150.0323;125.0246	0.77	quercetin hydrogenation	+				+		+	+		/
M7	11.34	C_16_H_12_O_7_	[M-H]^−^	315.0513	315.051	0.95	300.0253;283.0261;271.0204;255.0265;243.0289;163.0027;151.0026;107.0131	1.95	methylated quercetin (isorhamnetin)	+	+	+	+	+	+	+	+	+	
M8	9.75	C_16_H_12_O_7_	[M-H]^−^	315.0515	315.051	1.59	300.0253;283.0261;271.0204;243.0289;163.0027;151.0026;107.0131	1.75	methylated quercetin	+		+	+			+	+		Du et al. (2017)
M9	10.25	C_16_H_12_O_8_	[M-H]^−^	331.0464	331.0459	1.51	316.0222;299.0184;271.0255;178.9989;164.0117;151.0037;124.0170;107.0144	1.40572	methylated myricetin		+	+	+	+	+	+	+	+	Lin et al. (2012)
M10	9.68	C_15_H_10_O_10_S	[M-H]^−^	380.9927	380.9922	1.31	301.0346;273.0397;178.9984;151.0035;121.0293;107.0132	0.055	quercetin sulphate	+			+	+	+	+	+		Guo et al. (2010)
M11^a^	9.09	C_15_H_10_O_11_S	[M-H]^−^	396.9868	396.9871	−0.76	317.0279;271.0255;179.0009;151.0033;109.0270	−0.49	myricetin sulphate					+	+	+	+	+	Guo et al. (2010)
M12^a^	*8.1*	C_21_H_18_O_12_	[M-H]^−^	461.0732	461.0725	1.52	285.0410;257.0407;229.0595;217.0522;	0.14	dehydroxylated quercetin monoglucuronide									+	/
M13	9.15	C_21_H_18_O_13_	[M-H]^−^	477.0675	477.0674	0.21	301.0338;255.0296;178.9978;151.0022	−0.61	quercetin monoglucuronide	+	+	+	+		+	+	+	+	Xue et al. (2011a)
M14	8.54	C_21_H_18_O_13_	[M-H]^−^	477.0677	477.0674	0.63	301.0338;271.0243;255.0296;178.9978;151.0022	−0.81	quercetin monoglucuronide	+	+	+	+		+	+	+	+	Xue et al. (2011a)
M15	9.05	C_22_H_22_O_12_	[M-H]^−^	477.1044	477.1038	1.26	315.0507;271.0224;243.0253;199.0416;151.0026	0.11	methylated hyperoside		+	+	+		+	+			Vacek et al. (2012)
M16	8.85	C_21_H_20_O_13_	[M-H]^−^	479.0805	479.0831	−5.43	479.0828;317.0308;299.0202;271.0239;178.9984;166.9990;151.0045;139.0042	−0.82	gossypin								+	+	Xue et al. (2011b)
M17	8.36	C_21_H_20_O_13_	[M-H]^−^	479.0814	479.0831	−3.55	479.0813;316.0223;287.0203;271.0269;259.0278;179.0012	−1.00	myricetin-3-glucoside				+			+	+	+	Du et al. (2017)
M18	9.19	C_22_H_20_O_13_	[M-H]^−^	491.0828	491.0831	−0.61	315.0512;300.0294;227.0254;151.0034;113.0238	−0.13	methylated quercetin monoglucuronide		+	+	+			+	+	+	Guo et al. (2010)
M19	9.05	C_22_H_20_O_13_	[M-H]^−^	491.0861	491.0831	6.11	315.0508;300.0272;255.0295;113.0215	−0.25	methylated quercetin monoglucuronide	+	+	+	+		+	+	+	+	Guo et al. (2010)
M20	8.96	C_22_H_20_O_13_	[M-H]^−^	491.0828	491.0831	−0.61	315.0512;300.0294;227.0254;151.0034;113.0238	−0.37	methylated quercetin monoglucuronide	+	+	+	+		+	+	+	+	Guo et al. (2010)
M21	8.85	C_22_H_20_O_13_	[M-H]^−^	491.0861	491.0831	6.11	315.0508;300.0272;255.0295;113.0215	−1.15	methylated quercetin monoglucuronide	+			+		+	+	+	+	Guo et al. (2010)
M22^a^	8.31	C_21_H_18_O_14_	[M-H]^−^	493.0617	493.0623	−1.22	317.0200;178.9928;150.9972;137.0207	−1.16	myricetin monoglucuronide				+			+			Lin et al. (2012)
M23^a^	8.79	C_22_H_20_O_14_	[M-H]^−^	507.0776	507.0780	−0.79	331.0331;316.0223;287.0206;271.0198;178.9993;164.0106;151.0052	−0.93	methylated myricetin monoglucuronide			+	+		+	+		+	Guo et al. (2010)
M24^a^	9.4	C_22_H_20_O_14_	[M-H]^−^	507.078	507.078	0.00	331.0461;316.0225;165.9911	−0.85	methylated hibifolin			+	+	+	+	+	+		Guo et al. (2010)
M25	7.64	C_21_H_20_O_15_S	[M-H]^−^	543.0449	543.0445	0.74	380.9929;301.0363;187.9774;178.9989;151.0034;79.9588	−1.79	hyperoside sulfate				+	+	+	+	+	+	Guo et al. (2011b)
M26	8.36	C_21_H_20_O_15_S	[M-H]^−^	543.0452	543.0445	1.29	463.0932;301.0404;271.0204;255.0363	−1.45	quercetin-3'-glucoside sulphate				+	+	+	+	+		/
M27	8.29	C_21_H_18_O_16_S	[M-H]^−^	557.0244	557.0242	0.36	477.0701;380.9865;301.0357;151.0031	−1.93	quercetin monoglucuronide sulphate	+	+		+	+	+	+	+	+	Lee et al. (2012)
M28	6.64	C_27_H_28_O_18_	[M-H]^−^	639.1208	639.1202	0.94	463.0880;300.0294;271.0208;243.0381	−2.45	hyperoside monoglucuronide				+			+		+	Guo et al. (2011b)
M29	7.47	C_27_H_28_O_18_	[M-H]^−^	639.1213	639.1202	1.72	463.0721;300.0176;271.0151;245.0044;211.9927;150.9989	−2.22	quercetin-3'-glucoside monoglucuronide	+			+		+	+		+	/
M30	7.96	C_27_H_26_O_19_	[M-H]^−^	653.1042	653.0995	7.20	477.0643;315.0521;301.0342;151.0033	−2.93	quercetin di-glucuronide	+	+		+			+		+	Lee et al. (2012)
M31	8.13	C_27_H_26_O_19_	[M-H]^−^	653.1042	653.0995	7.20	477.0643;315.0521;301.0342;151.0033	−2.69	quercetin di-glucuronide	+	+	+	+		+	+		+	Lee et al. (2012)
M32	7.94	C_28_H_30_O_18_	[M-H]^−^	653.1369	653.1334	5.36	491.0755;477.0894;315.0490;300.0286;150.9994	−1.98	methylated hyperoside monoglucuronide	+	+		+		+	+		+	Guo et al. (2011b)
M33	8.07	C_28_H_30_O_18_	[M-H]^−^	653.1372	653.1334	5.82	491.0755;477.0894;315.0490;300.0286;150.9994	−1.89	methylated hyperoside monoglucuronide	+	+	+	+		+	+		+	Guo et al. (2011b)
M34^a^	8.69	C_27_H_28_O_19_	[M-H]^−^	655.1154	655.1152	0.31	479.0727;475.0527;431.0618;387.0343;317.0306;166.9988;139.0017	−2.94	gossypin monoglucuronide					+	+	+	+	+	/
M35^a^	7.9	C_27_H_26_O_20_	[M-H]^−^	669.0942	669.0944	−0.30	493.0653;317.0331;299.0208;273.0455;227.0366;194.9963;166.9996;139.0065	−4.17	hibifolin monoglucuronide							+		+	/
M36^a^	8.26	C_27_H_26_O_20_	[M-H]^−^	669.0948	669.0944	0.60	493.0459;317.0195;178.9918;150.9997	−3.49	myricetin di-glucuronide				+		+	+		+	Lin et al. (2012)
M37^a^	8.07	C_27_H_26_O_20_	[M-H]^−^	669.0948	669.0944	0.60	493.0653;317.0331;299.0208;273.0455;227.0366;194.9963;166.9996;139.0065	−3.66	hibifolin monoglucuronide							+		+	/
M38^a^	8.08	C_31_H_37_N_3_O_18_S	[M-H]^−^	770.1793	770.1720	9.48	752.1652;676.1203;463.0883;301.0389;271.0345;151.0030	−3.72	hyperoside glutathione conjugation	+	+	+	+		+			+	/

^a^ unreported new metabolites; Se, H, Li, K, Je, Co, Je_N, Co_N, and U represent mouse serum, heart, liver, kidney, jejunum, colon, jejunal contents, colonic contents, and urine samples, respectively.

#### 3.4.1 Quercetin-related prototypes and metabolites

Metabolite identification relied on the parent compound’s molecular formula and fragmentation pattern in MS. As shown in Supplementary Figure S1A, quercetin showed [M-H]^-^ *m/z* 301.0354 (C_15_H_9_O_7_^−^, ppm −2.3) at 10.5 min in XIC. In MS/MS spectrum, the first step of the fragmentation of quercetin involved Retro-Diels-Alder (RDA) rearrangement through the 1,2 site on the C-ring, which generated the product ion at *m/z* 178.9986 (1,2A^−^), then the ion at *m/z* 151.0037 was generated through the loss of CO from *m/z* 178.9986. Besides, the fragmentation ion at *m/z* 107.0139 was generated via RDA rearrangement on 0,4 site of the C-ring. Simultaneously, the loss of CH_2_O resulted in the formation of the product ion at *m/z* 273.0405.

M7 and M8 eluted at 11.34 min and 9.75 min, showed [M-H]^-^ at *m/z* 315.05 (0.95 ppm) with molecular formula C_16_H_12_O_7_ in MS spectrum. M7 and M8 exhibited a mass increase of 14 Da compared to quercetin, which suggested they might be methylation metabolites of quercetin. In the MS^2^ spectrum, the molecular ion at *m/z* 315.05 first removed CH_2_ to produce the ion at *m/z* 317.03. The latter ion undergoes RDA rearrangement through 1,3 sites on the C-ring to generate *m/z* 151.00 (1.3A^−^). Besides, the fragmentation ion at *m/z* 107.01 was obtained by RDA rearrangement on 0,4 site from *m/z* 301.04. The fragmentation pattern observed in M7 was in agreement with that of the isorhamnetin standard compound. Hence, M7 and M8 were tentatively speculated to be isorhamnetin and its isomer.

#### 3.4.2 Myricetin-related prototypes and metabolites

As shown in Supplementary Figure S1B, myricetin showed [M-H]^-^ at *m/z* 317.03 (C_15_H_9_O_8_^-^, ppm 3.47) at 9.32 min in XIC. In MS/MS spectrum, myricetin first underwent RDA rearrangement through 1,3 sites on the C-ring, which generated the ion at *m/z* 151.00 (1,3A^−^), then the ion at *m/z* 123.01 was generated by the elimination of CO from 1,3A^−^. Meanwhile, the fragmentation ion at *m/z* 178.99 was obtained by RDA rearrangement on 1,2 sites from myricetin. Besides, myricetin underwent a loss of CH2O2 to generate the product ion at *m/z* 287.02. Further deoxygenation of the latter produced the ion at *m/z* 271.03.

M4 and M5 eluted at 9.71 and 8.24 min, showed [M-H]^-^ at *m/z* 301.04 (−0.66 ppm) with molecular formula C_15_H_10_O_7_ in MS spectra. In MS^2^ spectra, the characteristic fragments of M4 and M5 were *m/z* 178.99, 151.00, 107.01 in negative, which fit with the characteristic product ions of quercetin. Compared with myricetin, M4 and M5 at *m/z* 301.04 were 16 Da less than myricetin, which indicated that the hydroxyl group was lost in B-ring from myricetin. Finally, we deduced that M4 and M5 were 3′-dehydroxylated myricetin and 4′-dehydroxylated myricetin, respectively by comparing the Clog P values (Table 3).

#### 3.4.3 Hibifolin-related prototypes and metabolites

As shown in Supplementary Figure S1F, the hibifolin showed [M-H]^-^ *m/z* 493.06 (C_21_H_19_O_13_^−^, ppm −5.35) at 9.2 min in XIC. In MS/MS spectrum, hibifolin first removed the glucuronide group to produce gossypetin (*m/z* 317.03). Then the gossypetin underwent neutral loss C_7_H_8_O_2_, which generated the product ion at *m/z* 194.99, then the ion at *m/z* 166.99 obtained via the removal of CO from *m/z* 194.99. Besides, the fragmentation ion at *m/z* 271.03 was generated via the removal of CH_2_O_2_ from *m/z* 317.03.

The retention time of M35 was 7.9 min. Its deprotonated ion was at *m/z* 669.09 (C_27_H_26_O_20_, -0.30 ppm), exhibiting a 176 Da higher mass than hibifolin. The ion with *m/z* of 493.06 was formed through the elimination of a glucuronic acid moiety from hibifolin. The characteristic product ions of hibifolin at *m/z* 317.03, 299.02, 194.99, and 166.99 were also presented in the MS^2^ spectrum of M35. Hence, M35 was identified as the mono-glucuronidation metabolite of hibifolin.

### 3.5 Relative quantification of flavonoid-related metabolites in db/db mice

**FIGURE 6:**
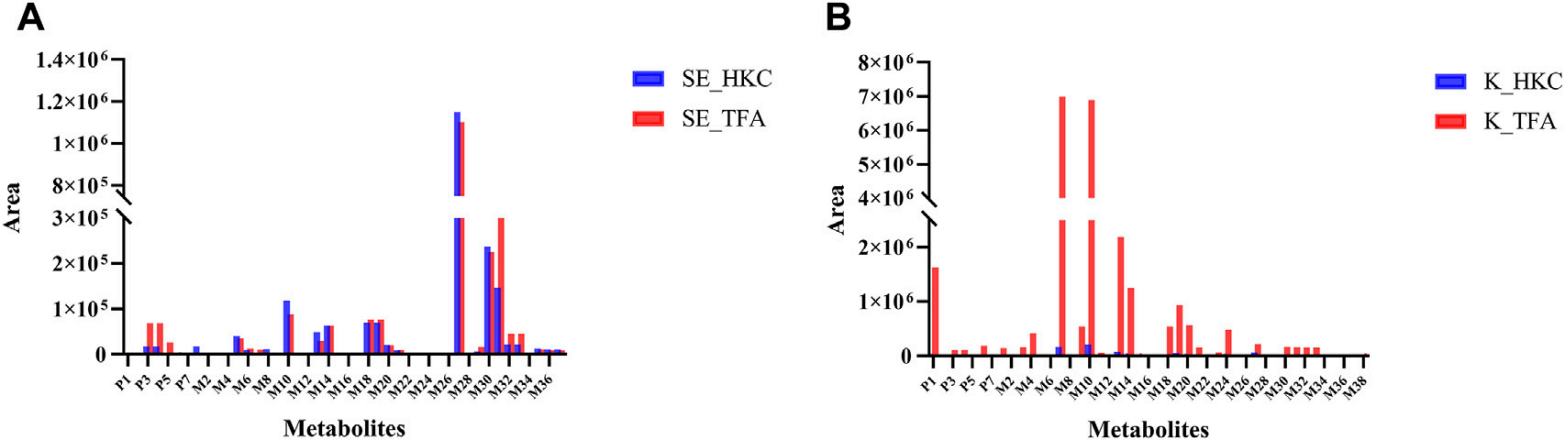
Relative quantification of flavonoid-related metabolites in serum and kidney of db/db mice after oral administration of HKC or TFA Relative quantification of flavonoid-related metabolites in serum **(A)** and kidney **(B)** of db/db mice after oral administration of HKC or TFA. SE, serum; K, Kidney; HKC, Huangkui capsule; TFA, total flavonoids of *Abelmoschus manihot* (L.) Medik.

The peak areas of the prototypes and metabolites corrected by internal standards were analyzed for relative quantification using Multiquant software to overcome the difficulty of quantifying metabolites due to the lack of standards. In the serum samples of db/db mice, 3 prototypes in descending order of exposure, i.e., hyperoside, isoquercitrin, and quercetin-3′-glucoside were detected. The main metabolic pathways were glucuronidation, sulfation, methylation, and glycosylation, then the relative quantification of metabolites in serum was performed. The highest exposure in the serum samples of db/db mice, however, was quercetin monoglucuronide sulfate as shown in Figure 6A. In the kidneys of db/db mice, 4 prototypes were detected. They were also presented in descending order of exposure: quercetin, hibifolin, hyperoside, isoquercitrin. The main metabolic pathways were sulfation, methylation, glucuronidation, dehydroxylation, and glutathione binding, then the relative quantification of metabolites in the kidney was performed. As shown in Figure 6B, the highest exposures in the kidney were quercetin sulfate and isorhamnetin (methylated quercetin). The result of the metabolites identified in other biological samples from db/db mice, and their relative quantification are represented in Supplementary Figure S2.

## 4 Discussion

In the present study, we have demonstrated that TFA has similar effects as HKC in treatment of db/db mice with DN. Based upon the experimental data, we have applied a comprehensive metabolite analysis strategy to systematically identify the main constituents and metabolites in serum, intestinal contents, urine, kidney, heart, liver, jejunum, and colon tissues of the db/db mice after oral administration of HKC or TFA.

Mass defect filter has been widely used to screen metabolites by imposing preset criteria around mass defects in selected core substructures (Liang et al., 2011). In general, the mass defect of the metabolite relative to the parent drug is always within 50 mDa, which helps automatically exclude unrelated ions from the complex matrix beyond the defined window (Hu et al., 2022). The combination of MDF and IDA acquisition method facilitates the detection of low-intensity metabolites associated with core structure. In this study, there are three core substructures among seven major flavonoids in TFA, namely, quercetin, myricetin, and gossypetin. Therefore, we used these three core substructures and their three metabolites: methylation, glucuronidation, and sulfation as MMDF templates to establish an IDA acquisition method that helped to mine all low-level flavonoid-related metabolites. Meanwhile, the combination of XIC, MDF, and PIF of Metabolitepilot software allowed us to quickly screen out all possible metabolites. Based upon this metabolite identification strategy, we have identified a total of 38 metabolites, including metabolic reactions such as dehydroxylation, deglycosylation, hydrogenation, methylation, glucuronidation, and sulphation, and corresponding recombination reactions. It is reported that the main metabolized forms of flavonoids in *A. manihot* (L.) Medik in normal rats are methylation and glucuronidation after deglycosylation. In contrast, nine new metabolites were identified in our study, which were for the first time reported. As listed in Table 3, 17, 16, 30, 16, 24, 12, 30, 23, and 35 metabolites were detected in serum, liver, kidney, heart, jejunum, colon, jejunal contents, colonic contents, and urine samples of db/db mice, respectively.

Due to the difficulty in obtaining the standards of many *in vivo* metabolites, we performed semi-quantification of all identified constituents and metabolites based on their peak areas after correction by internal standards. The results of the semi-quantification indicated that the metabolites quercetin monoglucuronide sulfate, quercetin di-glucuronide, quercetin sulfate, and isorhamnetin monoglucuronide were widely present in the serum of db/db mice, and the exposure of the prototype quercetin and the metabolites isorhamnetin, quercetin sulfate, quercetin monoglucuronide, and isorhamnetin monoglucuronide were higher in kidney of db/db mice after long-term administration of TFA. Therefore, these metabolites may play a key role in TFA treatment of DN.

Previous studies have reported that quercetin may enhance renal function and ameliorate renal oxidative stress levels and inflammatory responses in DN models (Lu et al., 2018; Ma et al., 2022). Isorhamnetin is a flavonoid glycoside element, which is abundant in herbal plants and has antioxidant activity for the treatment of cardiovascular diseases (Gong et al., 2020), but its therapeutic effect on DN has not been reported. Similar to what we have found in the present study, the glucuronide-sulfate conjugate of quercetin and isorhamnetin has a significantly higher *AUC* than the flavonoid aglycone when taken orally with the ethanol extract of *A. manihot* (L.) Medik (Guo et al., 2016). Thus, these conjugates may contribute to the efficacy of *A. manihot* (L.) Medik. It is reported that the glucuronide-sulfate conjugate of quercetin and isorhamnetin can inhibit active oxygen-related inflammation (Terao et al., 2011), reduce blood pressure (Galindo et al., 2012), and inhibit tumor proliferation (Delgado et al., 2014). However, the renal protective activity of these conjugates has not been reported. As the main metabolites of TFA in db/db mice, the pharmacological activities and mechanisms of these conjugated metabolites need to be further investigated. In addition, we found that the metabolite 3,4-dihydroxyphenylacetic acid (DOPAC), which is reported to be produced by quercetin in the presence of intestinal bacteria, has antioxidant effects and is less cytotoxic than quercetin (Tang et al., 2016). Besides, DOPAC has the effect of regulating glucose homeostasis in proximal renal tubular NRK-52E cells (Álvarez-Cilleros et al., 2018). Therefore, DOPAC may contribute to the pharmacological effect of TFA on DN.

In general speaking, chronic kidney disease can lead to a breakdown of intestinal barrier function, which may increase the absorption of uremic toxins such as indolyl sulfate and para cresol sulfate. These uremic toxins are formed via sulfotransferases (SULTs) (Watanabe et al., 2013). Flavonoids and their conjugated metabolites have been reported to inhibit SULTs activity (Wong and Williamson, 2013). Therefore, the flavonoid conjugates inhibit uremic toxin production, which may be one of the possible mechanisms of TFA in treating DN. Furthermore, in the present study, we found that both HKC and TFA groups had significant effects in reduction of albuminuria while the exposures of flavonoids and metabolites in peripheral blood between these two groups might not be the same. Therefore, the flavonoids of *A. manihot* (L.) Medik. may act through regulation of intestinal bacteria and correlation of metabolites but not primarily in kidneys. Besides, it was reported that the interaction among flavonoids, phase II enzymes, and efflux transporters affects the disposal of flavonoids and their metabolites (Jiang and Hu, 2012). Therefore, further investigation on whether other classes of metabolites in HKC can influence the distribution and disposal of flavonoid prototypes and metabolites in kidneys has been taken into our consideration.

In summary, we have for the first time applied a comprehensive metabolite identification and analysis strategy to systematically describe the exposure components and metabolic profiles of flavonoids in *A. manihot* (L.) Medik in db/db mice. A total of 38 metabolites were identified to be related to the flavonoids of *A. manihot* (L.) Medik, while quercetin, isorhamnetin, quercetin sulphate, quercetin monoglucuronide and isorhamnetin monoglucuronide were found to have high exposure in serum and kidneys of db/db mice. Therefore, the present study provides useful information for a better understanding of the therapeutic metabolites of the flavonoids of *A. manihot* (L.) Medik and their pharmacological mechanisms in treatment of DN. However, there are limitations to our study. This is an early-stage exploratory study on the efficacy of TFA in db/db mice. There is only one dose for HKC and TFA because of the complexity of the intervention. Further efficacy and mechanistic studies are needed in the future.

## Data Availability

The original contributions presented in the study are included in the article/Supplementary Material, further inquiries can be directed to the corresponding authors.
