# Supplementary figures and images for: Identification of the main flavonoids of Abelmoschus manihot (L.) medik and their metabolites in the treatment of diabetic nephropathy
Source: Front Pharmacol. 2024 Jan 8;14:1290868. doi: 10.3389/fphar.2023.1290868 (PMC10836608; doi:10.3389/fphar.2023.1290868)

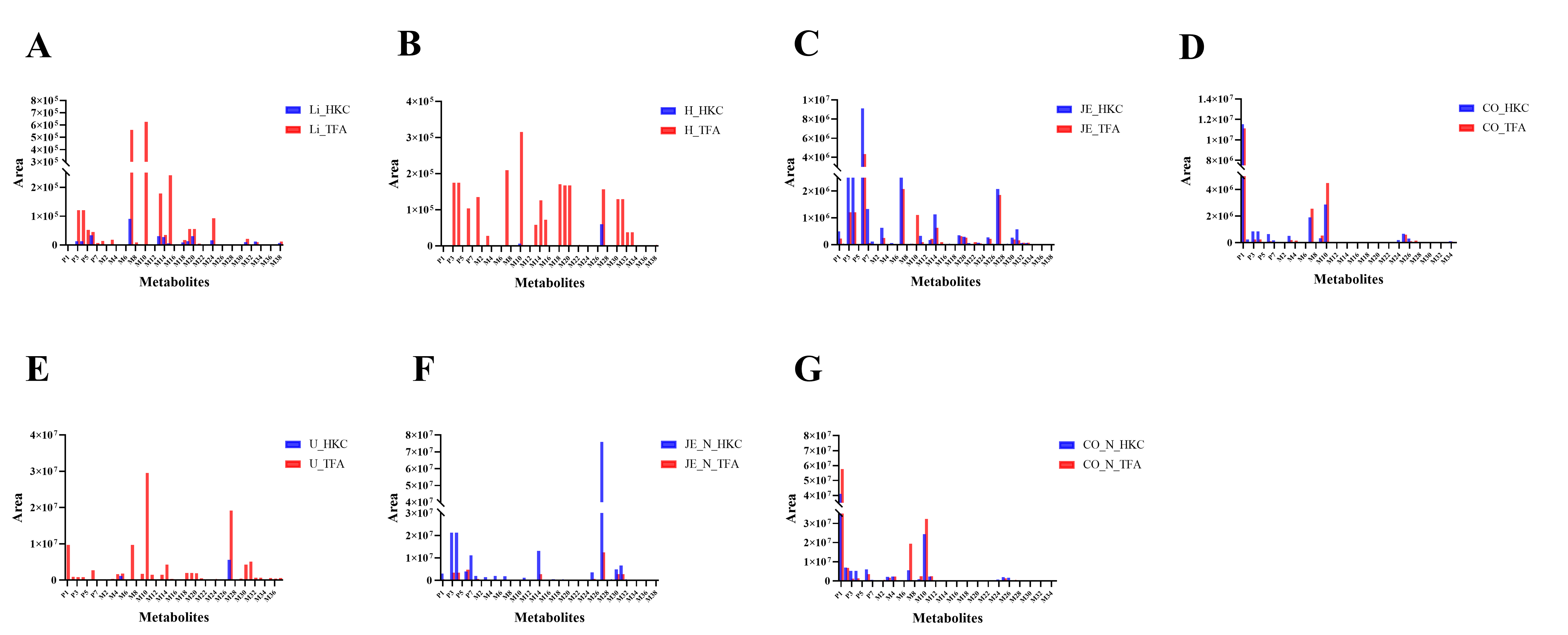

Supplement: Supplementary file 1 [file Image2.TIF]

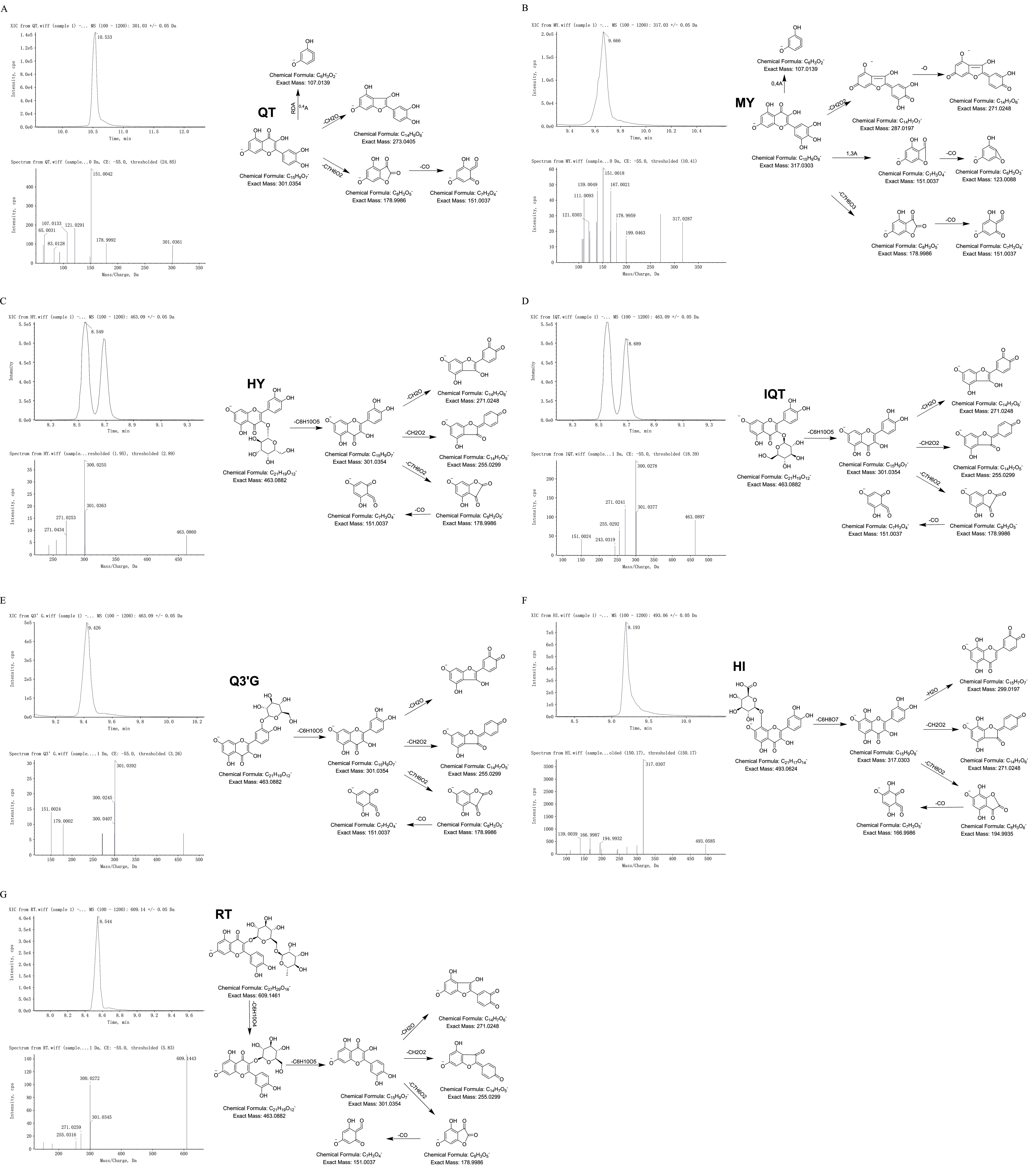

Supplement: Supplementary file 2 [file Image1.TIF]
